# Acquisition of Ownership Illusion with Self-Disownership in Neurological Patients

**DOI:** 10.3390/brainsci10030170

**Published:** 2020-03-15

**Authors:** Mariella Pazzaglia, Anna Maria Giannini, Francesca Federico

**Affiliations:** 1 Department of Psychology, Sapienza University of Rome Via dei Marsi 78, 00185 Rome, Italy; annamaria.giannini@uniroma1.it; 2 IRCCS Fondazione Santa Lucia, Via Ardeatina 306, 00179 Rome, Italy; 3 Department of Social and Developmental Psychology, Sapienza University of Rome Via dei Marsi 78, 00185 Rome, Italy; francesca.federico@uniroma1.it

**Keywords:** disownership, ownership, tumor resection, disembodiment, parietal cortex, spinal cord injury, rubber hand illusion, neuroplasticity, body representation

## Abstract

The multisensory regions in frontoparietal cortices play a crucial role in the sense of body and self. Disrupting this sense may lead to a feeling of disembodiment, or more generally, a sense of disownership. Experimentally, this altered consciousness disappears during illusory own-body perceptions, increasing the intensity of perceived ownership for an external virtual limb. In many clinical conditions, particularly in individuals with a discontinuous or absent sense of bodily awareness, the brain may effortlessly create a convincing feeling of body ownership over a surrogate body or body part. The immediate visual input dominates the current bodily state and induces rapid plastic adaptation that reconfigures the dynamics of bodily representation, allowing the brain to acquire an alternative sense of body and self. Investigating strategies to deconstruct the lack of a normal sense of bodily ownership, especially after a neurological injury, may aid the selection of appropriate clinical treatment.

## 1. Introduction

A normal awareness of one’s own body and body parts is essential for perception–motor interactions and is fundamentally critical for cognition. Patients with right frontal-parietal brain lesions sometimes disown their left limb [1]. After experiencing a spinal cord injury (SCI), patients may question the completeness of and sense of ownership over their own body [2]. After a right temporal-parietal cortex tumor resection, a patient experienced a sense of complete disownership of their entire physical body [3]. People with such neurological conditions grapple with an unclear sense of “mineness” pertaining to their own bodies. 

The feeling of body ownership—the sense of fundamentally possessing one’s body and self—is presumably the product of a complex system of multisensory awareness that effortlessly creates a unified, coherent experience of one’s self and body, including real-time updates [4]. Ownership of actual body parts or the body as a whole can be dissociated after brain and spinal cord damage [1,5] as well as during experimental manipulations of self-attribution by using a virtual body and multisensory conflict [6]. Frontoparietal and insular cortices integrate multisensory perceptions that generate the feeling of body ownership [7,8,9,10]. Injury, malfunction, or altered neural information from and toward these cortices may generate errors in self-attribution. Modification of self-attribution can also be easily induced in healthy research subjects, as in the “rubber hand illusion” (RHI), a renowned experimental paradigm that alters the congruity of multisensory information and manipulates the sense of body ownership [6]. An illusory ownership could be induced by experimentally generating conflict during the integration of multisensory bodily information. Through temporal congruency of vision, touch and proprioception, it is possible to feel artificial body parts or an entire virtual body as one’s own body. During this experimental procedure, synchronous (but not asynchronous) touching of a visible rubber hand and the real hand, which is hidden from view, induces a temporary feeling of ownership over a fake hand. This attribution is typically measured by objective (the perceived position of the real hand toward the rubber hand) and subjective (the experience of owning the rubber hand) indices. While some participants report subjective experiences such as the disappearance of their real hand, a sense of loss of its position and that it is no longer part of their own bodies, others indicate that this loss appears to be relatively rare, or at least very weak during RHI [11,12,13,14]. The complex neurobiological model underlying this sense of body ownership has been systematically investigated in neurologically intact individuals; however, studies in patients with neurological lesions can shed considerable light on how the sense of body ownership is generated. Here, we focus on the problem of body ownership from the clinical perspective, focusing on perceptual and neural similarities in selected neurological conditions where loss of ownership has been experimentally investigated through multisensory impressions and integration. In particular, we will discuss how loss of disownership can be experimentally achieved by using the RHI [6]. We suggest that multisensory stimulation may aid the development of appropriate clinical treatment in neurologic patients with altered sensory information and/or a disruption of body ownership perception.

## 2. Multisensory Network Codes for Body Ownership

Neuroimaging studies have demonstrated that the sense of bodily self-consciousness involves a multisensory integration of input that is realigned and integrated in a unique body-centered reference frame in the frontoparietal and temporal-parietal regions [4,15]. In particular, the parietal lobe in the right hemisphere is central to the representation of various parameters of bodily knowledge [16]. Right temporal-parietal lesions induce selective deficits in the representation of spatial relationships between various body parts and the whole body [17] as well as in the structural/topological knowledge of body unity [18,19]. An illusory, visual localization of the self into extrapersonal space (an out-of-body experience) has been reported in patients with right temporal-parietal damage [20]. However, dynamic changes in generating and updating the feeling of limb ownership are centered in frontoparietal regions, including the frontal premotor structures [21]. Therefore, inferior frontal lesions lead to disorders of body awareness and movement representations. Patients who exhibit disownership show additional damage in subcortical structures: thalamus, basal ganglia, and the surrounding white matter [22]. 

Hemodynamic markers of brain activity are related to feelings of body ownership during RHI in healthy individuals, further confirming that the illusion of body ownership depends on multisensory spatial and temporal congruence in the premotor cortices and along the intraparietal sulcus and inferior parietal cortex [23,24,25]. Frontoparietal areas, which are strongly interconnected, reflect changes in self-identification and self-location toward the position of the virtual body [4]. Neuroimaging studies have also suggested that vestibular representation in the right posterior insula is critical for integrating the body position in space and for maintaining the sense of ownership that follows illusion [26]. Of note, other cortical areas, such as the extrastriate body area, have been implicated in the distinction between the visual, proprioceptive, and tactile information involved in the experience of owning a limb [8]. Recent studies demonstrate that such bizarre experiences result from an altered functional connectivity among these body-related cortical regions and the basal ganglia or cerebellum, indicating that even subcortical pathways support the optimization of the crossmodal prediction of temporal and spatial signals, contributing to the cortical multisensory processing that forms a coherent, higher-level representation of the body [4]. 

Experiences of body ownership misperception appear to be related to an altered multisensory convergence of neural signals in the fronto-temporal-parietal cortex, where signals are significantly modulated by corticospinal projections. This observation indicates that reducing this network’s activity after a tumor resection or stroke may impact bodily self-consciousness [27]. In view of this information, we expected that even reduced peripheral inputs, which alter and diminish one’s somatic and motor control of their real hand, would influence the perception of body ownership. Indeed, in cases with SCI or brachial avulsion, in the absence of cerebral lesion, alterations of efferent (motor) and afferent (sensory) information, resulting in major changes in multisensory integration, can impact the capacity to acquire a sense of ownership over a surrogate body [28,29]. Indeed, a deafferented/deefferented part of the body reduces proprioceptive tactile and motor control to correctly “locate” the true hand.

## 3. Illusory Ownership Alters Self-Embodiment in Neurological Patients 

In neurological patients, a disrupted sense of body and self, sense of disownership [30], and delusions of various body parts [31] can be the consequences of a failure to realign continuous and coherent visual, tactile, proprioceptive, vestibular, and interoceptive signals that converge at the fronto-temporal-parietal and insular structures [32,33]. 

Recently, Smit and colleagues [3] reported an unusual case of a man who, after undergoing a right temporal-parietal cortex tumor resection, developed a lack of ownership over his entire body despite possessing intact somatosensory perception. To restore a sense of corporeal self-ownership in the patient, the researchers focused on two key, intriguing questions underlying this ownership. First, they assessed whether rapid and pronounced changes to illusory ownership of the patient’s body could be induced experimentally using RHI, which has been interpreted as implicit evidence for creating a successful surrogate embodiment [34,35]. Second, they investigated the interplay between incoming sensory signals from the brain, which alter the state of disembodiment. 

Applying a synchronous tactile stimulation to the left hand of the patient and to a rubber hand increased the patient’s sense of ownership over the rubber hand, as reflected by both outcome measures: an (objective) proprioception and (subjective) embodiment questionnaire. Not only was the illusion of body ownership induced, but when the patient looked at the rubber hand without receiving any tactile stimulation to the real hand, there was no difference between synchronous stroking and visual input for both measures, indicating that the subjective and objective judgments relative to illusion were essentially based on vision. Thus, evoked illusory ownership reveals that RHI is a powerful tool used to alter and capture the patient’s sense of body ownership. The patient’s heightened susceptibility to obtaining ownership over a virtual hand, despite actually sensing complete disownership of his entire body, is a result that may appear paradoxical. In this respect, previous studies in patients with brain damage have unraveled a sense of body and self that is more intense for the body parts that are inaccessible to awareness [21,32,36,37]. In the case of disownership, visual cues from a realistic body part can be sufficient to generate an illusory sense of ownership of the rubber hand in patients with a right frontoparietal lesion [1]. Even for patients with SCI and peripheral alteration of bodily signaling due to physical loss, the effect of the RHI (measured subjectively through questionnaires and objectively by the mislocalization of the sense of position between the actual and artificial body) was more evident in those patients who had greater loss of somatosensory sensation than in patients with intact perception [5]. Together, this indicates that neurological patients with a sense of disownership appear to be overwriting the disownership of their biological hand with visual location information from the rubber hand [38]. As observed in patients with specific neurological conditions [28,39,40,41,42], the brain may be more prone to incorporating artificial surrogates such as a realistic prosthetic hand into the representation of the individual’s own body [43] when the person’s normal sense of body is weak or absent. 

Furthermore, the extent to which there is a decrease in the reliability of ownership cues for one’s real hand increases proportionately with the subjective strength of the illusion, as indicated by the physiological measure of the RHI [41,42]. It has been surmised that healthy participants’ illusions of body ownership are experienced in tandem with the disownership of their real hand. Therefore, healthy participants never report the sensation of having three arms during RHI [12]. If the illusion is evoked, the rubber hand and the actual hand become unified, suggesting that if there is a sense of disownership of the intact, actual hand, the fake representation is experienced as the “replaced” hand [11,12]. In terms of physiological reactions, decreased ownership of the real hand can lead to reduced corticospinal excitability [44,45], increases in pain thresholds [46], histamine reactivity [47], slower tactile processing [48], and decreases in temperature [48] recorded in the real hand. Recognizing a body as belonging to oneself certainly plays a role in ascribing the weight of conflicting sensations, derived from the rubber hand, to the real hand, and one must subjectively resolve the initial conflict [11]. However, disownership of one’s own hand is not a prerequisite in RHI, and all the physiological changes to the real hand are highly questioned and debated in the literature [13,49,50]. 

## 4. The Multisensory Integration of Bodily Signals is Constrained by Somatic Perception

Disruption or instability in the bodily self seems to affect multisensory processes involving the affected limb, which suggests a strong influence of sensorimotor information over the longer term, experiential body representation. Cases of disownership after a stroke in the frontoparietal area display a robust visual capture of an illusionary ownership of the rubber hand without brushstrokes or other tactile stimulation [1,36]. Atypical susceptibility to the RHI, however, has been reported in Parkinson’s disease, in which patients experience more proprioceptive drift than the healthy controls under synchronous (illusion-inducing) as well as in asynchronous conditions (control), probably due to primary proprioceptive impairments [51]. Visual–tactile application of the RHI in individuals with SCI seems to elicit the reemergence of illusionary ownership when somatic sensation from the periphery is residual. These observations indicate that patients benefit more from vision “overruling” [52] and visual capture [53] to overcome the absence of somatic inputs than from the interplay of conflicting multisensory information. As hypothesized in other studies, many neurological patients may rely more on visual capture and less on proprioceptive and tactile cues than healthy subjects do because of the difficulty localizing the biological hand’s position using normal sensation [51,54]. Absent, attenuated, or incongruent somatosensory precision may increase a person’s capacity to “lose” their own hand and engender spatial remapping on the rubber hand as their own. 

Despite a significant number of studies on the impact of somatic information on normal multisensory integration of bodily signals, the cognitive and neural mechanisms underlying the visual capture of proprioception and its relation to a misperception of body ownership remain unclear. This includes the pertinent question of whether visual effects are limited to modulations of surrogate ownership or whether they extend to stronger, generative effects that evoke body perceptions. Smit and colleagues [3] reported a patient who displayed a pronounced sense of illusion after a right temporal-parietal cortex tumor resection, with visual exposure to the rubber hand without tactile manipulation. In this case, the heightened susceptibility to a body-ownership illusion cannot be explained by the altered processing of multisensory integration alone [3]. Despite a sense of complete disownership, the patient was reported to have intact somatosensory perception, motor planning, and execution [3]. 

Thus, while the benefit of vision is understandable in amputees where the biological segment is absent, the value of vision is less obvious when the biological body part is still anatomically and functionally present. The patient who “lost” his body after a tumor resection did so when his vision of his body was blocked, as is the case during the RHI. From the perspective of the brain, physical disembodiment and amputation [55] could be treated as similar when using the RHI paradigm: in both cases, given the inability to record the visual experience of one’s own physical body, alternative ownership has no effect on the natural experience of embodying one’s real body.

Accordingly, it is important to note that disownership effects can be generated along a continuum of variables. One way could be by decrementing the weight of the senses coming from the body (such as touch and proprioception in SCI and Parkinson disease); another way would be to experience a complete detachment from the physical body (such as when the patient “lost” his body or after frontoparietal lesions). In both of these cases, they could not feel or control their actual body. Under certain circumstances of visuo-tactile integration, patients may temporarily rely on a more visually based image of their body rather than on their somatosensation. This raises the possibility that the virtual body does not produce any multisensory conflicts or implicit violations of the expectations and representations which describe what one knows one’s body is truly like, facilitating the perceptual flexibility needed for the body ownership illusion [56]. However, evidence for the specific cognitive mechanisms underlying visual capture as it relates to the body ownership illusion is still lacking. An understanding of the numerous bodily signals in the brain is required to determine how the cerebral organization of the interconnected cortical areas, subcortical areas, and corticospinal projections contribute to the identification of corporeal awareness. In cases where neurological damage has disturbed the multisensory integration in the cortical and subcortical body areas, reducing the conflict between the visual, tactile, and proprioceptive inputs in the body, visual cues from a realistic body part can be sufficient for not only recalibrating the hand’s position, but also for generating feelings of ownership of the fake hand [1]. Even in healthy subjects, an attenuation of responses in the somatosensory cortex may be useful to resolve perceptual ambiguity about perceived ownership of the body [57,58]. Tentatively, we suggest that under clinical conditions, such an enhanced response to the RHI may be pivotal to altering the sense of body ownership, mediated by an increased dependence on visual cues and less on proprioceptive and tactile controls or body memory. Taken together, these neurological studies suggest that the visual capture effect may be due to a temporary disruption of any conscious and unconscious information (the weighting and integration of proprioceptive, tactile, interoceptive signals) or damaged top-down mechanisms (e.g., instability of the bodily self or altered signals in the frontoparietal cortex) of the participants’ body representation. The vision effect may further reduce the relative weight of somatosensory stimuli, leading to augmented subjective feelings of ownership for the rubber hand.

This neurological picture may help to reconcile the discrepancy between alternative models of body ownership that are not based on multisensory integration, [29] models in which multisensory interactions act at the initial stage to resolve conflicting perceptions in probabilistic representation and self-awareness based on predictive coding signals about the body.

## 5. Conclusions

In this analysis, we discussed the recent evidence on rapid experimental changes in body perception that occur under conditions of a weak or an absent sense of body ownership, pursuing their implications in unraveling the mechanisms underlying bodily perception. A close scrutiny of clinical populations during experimental manipulation of bodily illusions may further elucidate the existing knowledge on the sense of disownership of one’s own body. It is easier to capture the sense of ownership over a virtual body under the condition of disownership of oneself. The virtual body may be possessed more easily because ownership of the rubber hand obviates the need to mask one’s ownership of the real hand. For the phenomenon of altering self-perception and the sense of embodying one’s own body, the temporal and spatial congruency rule is not critical for determining the multisensory perception. Vision seems predominant in triggering the prediction, weighting, and elicitation of ownership sensations in RHI.

An empirically tractable and predictable model to understand bodily disownership has important implications for clinical and rehabilitative approaches to physical impairment and to improving the scientific understanding of the sense of “my body” in one’s mind. This finding suggests that when conceptualizing the patient’s condition, any level of physical disembodiment should be considered as not only a deficit but also a modification of both brain plasticity and the ability to embody a surrogate body part (for example, a rubber hand). Under certain clinical conditions, a realistic artificial hand could be an assistive tool or a surrogate for developing new rehabilitative strategies and for translational research [59,60,61].

Despite the lack of clarity about the mechanisms underlying illusory body ownership, recent studies have suggested that RHI has beneficial effects on attenuating pain [42,62] as well as possible residual somatic sensations [41]. Patients with neurological disorders may derive therapeutic benefits from coherent sensory stimulations of a virtual body that they perceive as their own.

Technological advancements in virtual-reality-based paradigms have also received significant clinical interest and are quickly being developed to offer solutions for restoring the subjective awareness, somatic illusion, and feelings of embodiment that are required to maintain a functional physical body [63,64].
